# Integrated Metabolomic and Transcriptomic Analysis Reveals Host Response Mechanisms to Porcine Epidemic Diarrhea Virus Infection in Pigs

**DOI:** 10.3390/vetsci13040313

**Published:** 2026-03-25

**Authors:** Yajing Zhou, Tongxi Lu, Jie Wang, Shanshen Gu, Ruihua Huang, Shenglong Wu, Wenbin Bao, Haifei Wang

**Affiliations:** 1 Key Laboratory for Animal Genetics, Breeding, Reproduction and Molecular Design, College of Animal Science and Technology, Yangzhou University, Yangzhou 225009, China; yajingzhoucindy@163.com (Y.Z.); lutongxilu@163.com (T.L.); wangjie4228@163.com (J.W.); gshanshen@163.com (S.G.); slwu@yzu.edu.cn (S.W.); wbbao@yzu.edu.cn (W.B.); 2 Institute of Swine Science, Nanjing Agricultural University, Nanjing 210095, China; rhhuang@njau.edu.cn

**Keywords:** PEDV, Large White pigs, transcriptomic, metabolomic, steroid hormone biosynthesis

## Abstract

Porcine epidemic diarrhea virus (PEDV) causes acute diarrhea, dehydration and death in piglets, resulting in significant economic losses in the pig industry. It is crucial to identify the pathogenesis and mechanism between host–PEDV interactions. In our study, we performed transcriptomic and metabolomic analyses in PEDV-infected Large White (LW) pigs. PEDV infection caused blunted and fused intestinal villi, necrosis of the intestinal mucosal epithelial cells and atrophy of intestinal glands. Transcriptomic and metabolomic analyses revealed 692 differentially expressed genes and 1485 differential metabolites, respectively. Among them, differentially expressed genes were enriched in virion assembly, lipoprotein metabolic process and PPAR signaling pathway. Differential metabolites were enriched in primary bile acid biosynthesis and lipoic acid metabolism. An integrated analysis of the transcriptome and metabolome revealed that differentially expressed genes and metabolites were co-enriched in steroid hormone biosynthesis and bile secretion. In addition, key metabolites Dehydroepiandrosterone (DHEA) and Estriol in steroid hormone biosynthesis both inhibited PEDV infection and alleviated the excessive inflammatory response in vitro. Collectively, our study constructed a multi-omics landscape of PEDV infection in LW pigs, providing potential targets for developing metabolic-targeted antiviral interventions.

## Simple Summary

Porcine epidemic diarrhea virus causes severe intestinal damage and high mortality in piglets. Identifying the pathogenesis and mechanism of host–PEDV interactions is important. This study investigated the molecular changes in Large White pigs during PEDV infection using a combination of transcriptomic and metabolomic analyses. Steroid hormone biosynthesis was identified as a critical pathway to regulate PEDV infection. In addition, metabolites Dehydroepiandrosterone (DHEA) and Estriol in steroid hormone biosynthesis inhibited PEDV infection in vitro. These findings provide a comprehensive map of PEDV infection and highlight potential metabolic targets for the development of new antiviral therapies.

## 1. Introduction

Porcine epidemic diarrhea (PED) is a highly contagious intestinal disease caused by the porcine epidemic diarrhea virus (PEDV). PEDV was first isolated in Belgium in 1977. It is an enveloped, single-stranded, positive-sense RNA virus classified into the genus Alphacoronavirus of the family Coronaviridae [1]. PEDV strains are generally classified into two different genogroups, including S INDEL strains (G1b), which contain multiple insertions and deletions in the S1 subunit of the S protein, and non-S INDEL strains (G2b), which are typically more virulent [2,3,4]. Since 2010, the outbreak of highly pathogenic variant strains has rapidly spread across multiple continents. Due to continuous viral mutation, existing vaccines have failed to provide complete protection, leading to a profound transformation in the global epidemiological patterns of the disease [5,6]. PEDV infection is characterized by clinical manifestations such as severe diarrhea, vomiting, anorexia and dehydration. Although PEDV can infect pigs at all developmental stages, it results in high mortality in suckling piglets, causing substantial economic losses to the pig industry [7]. The small intestine is an important part of the systemic immune system [8]. PEDV targets the small intestine, specifically infecting villous enterocytes and mesenteric lymph nodes. This leads to acute necrosis of epithelial cells and villous atrophy, causing malabsorption and watery diarrhea [9].

Transcriptome analysis is a valuable technology to reveal the molecular mechanisms driving development, disease and virus infection [10]. Metabolomics serves as a powerful approach for characterizing bioactive compounds and elucidating metabolic processes [11]. Viruses reprogram host metabolic phenotypes, such as upregulating nucleotide and lipid biosynthesis, to meet the anabolic demands of viral replication and propagation [12,13]. Viruses also employ diverse strategies to modulate metabolism to achieve their replication and propagation [14]. The integration of transcriptome and metabolome gives a comprehensive insight into understanding the mechanism of viral infections. Wu et al. revealed key immune and susceptibility differences, offering new insights into PRRSV resistance and control strategies via combining transcriptomic and metabolomic analysis [15]. Zhou et al. established a multi-omics landscape of the host response, providing a vital basis for understanding TGEV pathogenesis through an integrated transcriptomic and metabolomic analysis [16].

Numerous studies have focused on the pathogenesis and immune regulation of PEDV, the metabolic alterations associated with infection remain poorly understood. In our study, Large White pigs were used as a model to investigate the function of PEDV. Metabolomics and transcriptomics analyses were used to analyze the mechanism during PEDV infection in vivo. Differentially expressed genes and metabolites involved in PEDV infection were identified during PEDV infection. ‘Biosynthesis of cofactors’, ‘Steroid hormone biosynthesis’ and ‘Bile secretion’ were also enriched under PEDV infection in vivo. In addition, DHEA and Estriol in steroid hormone biosynthesis both inhibited PEDV infection and alleviated the excessive inflammatory response. The results provided the metabolomic and transcriptomic landscape of PEDV infection and contributed to our further understanding of PEDV pathogenesis.

## 2. Materials and Methods

### 2.1. Animal Experiments

All animal experiments were approved by the Institutional Animal Care and Use Committee of the Yangzhou University Animal Experiments Ethics Committee [permission number SYXK (Su) 2021-0026]. G2a subtype strain PEDV GX4/2021 (GenBank: OP382083) was reserved in our laboratory. Ten 3-day-old newborn LW pigs were obtained from a pig farm in Jiangsu province and housed in a specific pathogen-free animal facility. For assessing the efficacy of PEDV in vivo, newborn pigs were divided into a mock group (n = 5) and PEDV groups (n = 5). The PEDV group was orally administered 20 mL milk containing 2 mL of 10^4.5^ PFU/mL PEDV, while the mock group was orally administered 20 mL milk containing 2 mL of PBS as a control. Post-euthanasia, tissue samples were collected under veterinary guidance.

### 2.2. Cells and Virus

The porcine intestinal epithelial cells (IPEC-J2) and African green monkey kidney cells (Vero), along with PEDV were maintained in our laboratory. Cells were cultured in Dulbecco’s Modified Eagle Medium (DMEM, Gibco, CA, USA) containing 10% fetal bovine serum (FBS, Gibco, CA, USA) and 1% penicillin-streptomycin-Amphotericin B (Solarbio, Beijing, China).

### 2.3. Viral Infection

Cells were seeded in cell culture plates and cultured overnight in a 37 °C incubator with 5% CO_2_. After being washed twice with PBS, the cells were infected with PEDV at a multiplicity of infection (MOI) of 1. After incubation for 2 h, unbound viruses were removed and then maintained in serum-free DMEM with 2 µg/mL trypsin and collected at the specified time points post infection.

### 2.4. Paraffin Section Preparation and H&E Staining

Intestinal tissues were fixed in 4% paraformaldehyde at 4 °C. Tissues were dehydrated with ethanol and then embedded in paraffin. After being deparaffinized and rehydrated, sections were stained with hematoxylin solution for 5 min. Then, sections were dehydrated in gradient alcohol for 5 min and stained in eosin stain for 5 min. Finally, sections were counterstained in eosin solution for 5 min and imaged using an optical microscope (Motic, Xiamen, China). Villus height and crypt depth were analyzed using K-Viewer software (1.5.3.1).

### 2.5. RNA Extraction and qRT-PCR Assay

Samples were lysed in 1 mL Trizol reagent (Takara, Dalian, China). RNA was isolated following the manufacturer’s instructions and reverse-transcribed into cDNA using HiScript III RT SuperMix for qRT-PCR (Vazyme, Nanjing, China). qRT-PCR was performed using SYBR Green Premix Pro Taq HS qPCR Kit II (Accurate Biology, Changsha, China) according to the manufacturer’s protocol. Viral RNA was absolutely quantified by qRT-PCR using a standard curve generated from serial dilutions of a plasmid containing the viral M gene. Primer sequences used in the qRT-PCR assays are as Table S7.

### 2.6. Western Blot Analysis

Samples were lysed in 1 mL RIPA lysis buffer containing 10% protease inhibitor. The concentration of protein was measured using an Enhanced BCA Protein Assay Kit (Beyotime, Shanghai, China) following the manufacturer’s instructions. The lysates were denatured 10 min in 5× SDS-PAGE loading buffer in 95 °C. For the Western blot, the proteins were separated by SDS-PAGE and transferred to PVDF membranes (Millipore, Burlington, MA, USA). Membranes were blocked with NcmBlot blocking buffer (NCM, Suzhou, China) at room temperature for 20 min and then washed with TBS containing 0.2% Tween-20 (TBST) three times. We incubated membranes with PEDV N antibody (Youlong Biotech, Shanghai, China) or GAPDH antibody (Proteintech, Wuhan, China) at 4 °C for a whole night. After being washed with TBST three times, membranes were incubated with the goat anti-mouse IgG (CWBIO, Taizhou, China) at room temperature for 2 h and detected with enhanced chemiluminescence (ECL) (Abbkine, Wuhan, China).

### 2.7. Transcriptome Data Analysis

Four biological replicates in the mock group and the PEDV group were lysed in Trizol reagent respectively. Total RNA was extracted according to the manufacturer’s instructions and transcriptome sequencing on DNBSEQ-T7 was detected by BioMarker Co., Ltd. (Beijing, China). To obtain high quality clean data, reads containing adapters or with a base mass fraction lower than 20 were removed. Clean reads were aligned to Sscrofa11.1 genome using software HISAT2 (2.2.1) [17]. StringTie (2.2.3) [18] software was employed to assemble the aligned reads and reconstruct the transcriptome for subsequent analysis. Genes with a fragments per kilobase million (FPKM) [19] value greater than 1 were considered as expressed. DESeq2 (1.48.1) [20] software was utilized for differential analysis between the mock group and the PEDV group. Genes were considered differentially expressed with the threshold of |log2-fold change| ≥ 1 and an adjusted *p*-value ≤ 0.05.

### 2.8. Metabolomics Analysis

Intestinal tissue samples that weighed more than 50 mg and five biological replicates in the mock group and the PEDV group were prepared for metabolomics detection. BioMarker Co., Ltd. provided metabolites extraction and LC-MS/MS analysis. An Acquity I-Class PLUS (Waters, MA, USA) high-resolution mass spectrometer was employed to separate and detect metabolites. Raw data were processed using Progenesis QI software (2.0) for peak extraction and peak alignment. Peak identification was performed based on the online METLIN database integrated in Progenesis QI, public databases, and the BioMarker database. The KEGG (https://www.genome.jp/kegg/pathway.html, accessed on 4 March 2025), HMDB (https://hmdb.ca/, accessed on 4 March 2025) and LIPIDMaps (https://www.lipidmaps.org/, accessed on 4 March 2025) databases were used to annotate the identified metabolites. Metabolites were analyzed using Orthogonal Partial Least-Squares (OPLS). Metabolites were considered differential metabolites with the threshold of VIP ≥ 1, *p* < 0.05 and |log2-fold change| ≥ 1 between the two groups.

### 2.9. Integrated Network Analysis of Transcriptomics and Metabolomics

Differentially expressed genes and metabolites were analyzed using two-way orthogonal partial least squares (O2PLS) analysis. All differentially expressed genes and metabolites were uploaded to KEGG database (https://www.kegg.jp/kegg/pathway.html, accessed on 11 November 2025) [21] to identify enriched pathways. We focused on biological and signal transduction pathways that involved both the differentially expressed genes and metabolites. Pathways with a *p*-value ≤ 0.05 were considered significantly enriched.

### 2.10. Cytotoxicity Assays

Cells were seeded in 96-well plates at a density of 2000/well in DMEM containing 10% FBS and incubated overnight. Cell culture medium was replaced with DMEM containing 10% FBS and different concentrations of DHEA or Estriol. After treatment with the indicated time, each well was supplemented with 100 μL of Opti-MEM and 10 μL of Cell Counting Kit-8 (CCK-8; Abbkine, Wuhan, China) solution, followed by 1 h of incubation at 37 °C. The absorbance at 450 nm was measured using a Tecan Infinite 200 microplate reader (Tecan, Männedorf, Switzerland).

### 2.11. 50% Cell Culture Infectious Dose (TCID_50_) Assay

Vero cells were seeded in 96-well plates at a density of 1 × 10^4^/well in 100 µL of DMEM containing 10% FBS and 1% penicillin/streptomycin. Virus stocks were then serially diluted 10-fold (ranging from 10^−1^ to 10^−7^) in serum-free DMEM, and 100 μL of each dilution was added to the cells. Each dilution was tested with eight replicates in 96-well plates. Plates were incubated at 37 °C with 5% CO_2_ for 7 days. Cytopathic effects were observed daily, and TCID_50_ was calculated by using the Reed–Muench method.

### 2.12. Statistical Analysis

Data are presented as mean ± standard deviation (SD). Student’s *t*-test was used to compare the differences between two groups using IBM SPSS Statistics 22. *p*-values < 0.05 were considered statistically significant (* *p* < 0.05, ** *p* < 0.01, *** *p* < 0.001).

## 3. Results

### 3.1. Effects of PEDV Infection in LW Pigs

**Figure 1:**
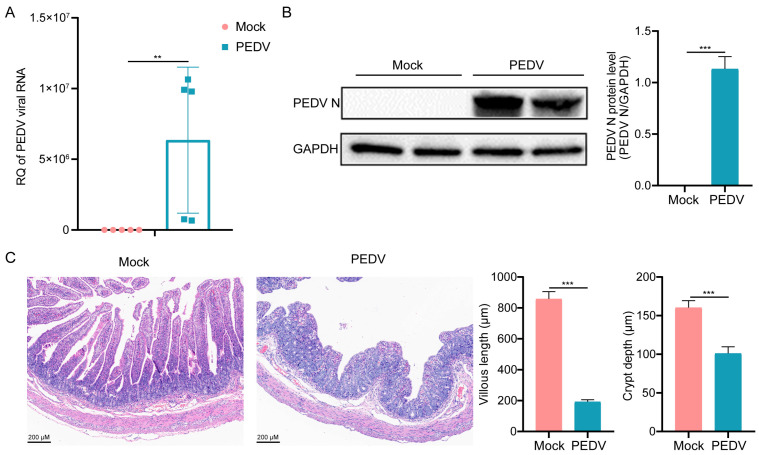
Effects of PEDV infection in LW pigs. (**A**) PEDV copy numbers were detected via qRT-PCR assay. (**B**) Western blot analysis of PEDV N protein. Right panel was the grayscale analysis of band intensities. (**C**) Hematoxylin and eosin (H&E) staining and statistical analysis of small intestinal morphology in small intestinal segment. Scale bar: 200 μm. Data are presented as the mean ± SD (n = 5). ** *p* < 0.01, *** *p* < 0.001.

To confirm successful infection in LW pigs, we investigated the viral load and protein expression. The qRT-PCR analysis and the Western blot analysis indicated high copy numbers of PEDV M and an abundant expression of PEDV N in the PEDV group respectively, while both were undetectable in the mock group (Figure 1A,B). To further explore the difference in intestinal structures between the mock group and the PEDV group, H&E staining was utilized to detect the intestinal mucosa of piglets. Compared to the mock group, the PEDV group exhibited blunted and fused intestinal villi, necrosis of the intestinal mucosal epithelial cells and atrophy of intestinal glands (Figure 1C). Collectively, these results demonstrated a successful PEDV infection in LW pigs, characterized by high viral loads and marked intestinal lesions, including mucosal damage.

### 3.2. Transcriptomic Analysis of PEDV-Infected Pigs

**Figure 2:**
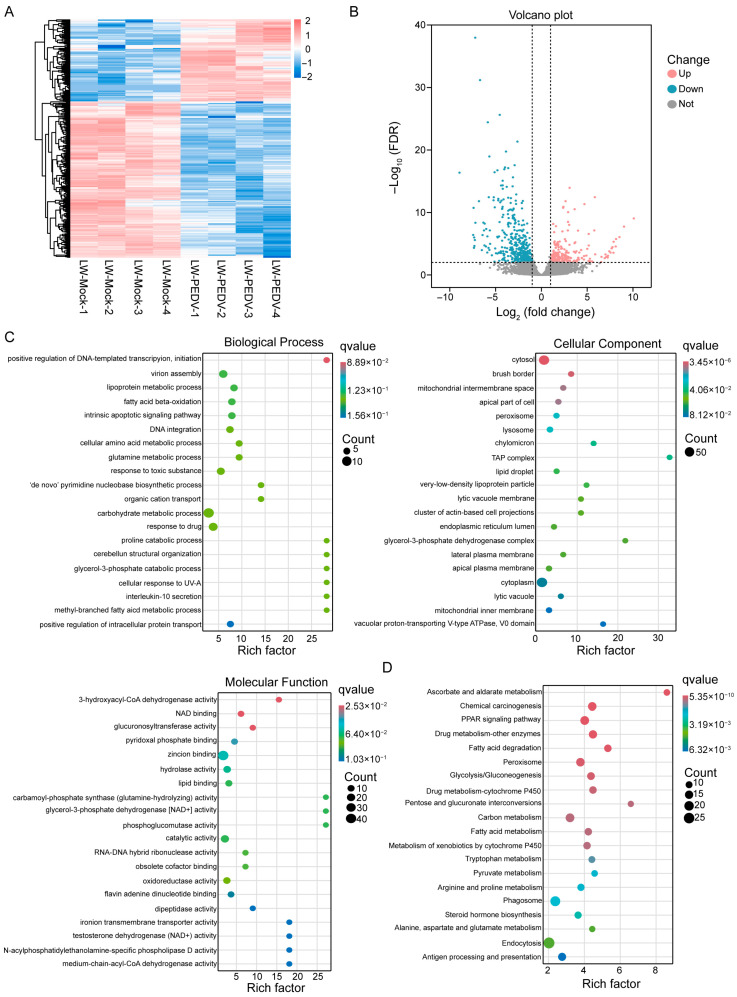
Transcriptomic analysis in mock and PEDV-infected groups. (**A**) Hierarchical clustering heatmap of differentially expressed genes between two groups. Blue represents downregulation and red represents upregulation. (**B**) Volcano plots of differentially expressed genes between two groups. Red and blue dots represent genes up-regulated and down-regulated, respectively. (**C**) GO analysis of DEGs was enriched in three pathway categories: biological process (BP), cellular component (CC), and molecular function (MF). (**D**) KEGG analysis of top 20 pathways between mock and PEDV-infected groups.

To identify potential regulatory mechanisms in response to PEDV infection, we performed RNA-seq analysis on intestinal tissues from both mock and PEDV-infected groups. Hierarchical clustering analysis was carried out to display the expression patterns of differently expressed genes (DEGs) between two groups (Figure 2A). Differential expression analysis identified 692 DEGs, of which 238 were up-regulated genes and 454 were down-regulated genes (Figure 2B, Table S1). Gene ontology (GO) enrichment analysis indicated that DEGs were significantly enriched in the biological process (BP), including in ‘positive regulation of DNA-templated transcription, initiation’, ‘virion assembly’ and ‘lipoprotein metabolic process’. For the cellular component (CC) category, enriched terms included ‘cytosol’, ‘brush border’ and ‘mitochondrial intermembrane space’, and molecular function (MF) terms primarily included ‘3-hydroxyacyl-CoA dehydrogenase activity’, ‘NAD binding’ and ‘glucuronosyltransferase activity’ (Figure 2C, Table S2). KEGG analysis revealed that DEGs were predominantly enriched in ‘Ascorbate and aldarate metabolism’, ‘Chemical carcinogenesis’ and ‘PPAR signaling pathway’ (Figure 2D, Table S3).

### 3.3. Metabolomic Analysis of PEDV-Infected Pigs

**Figure 3:**
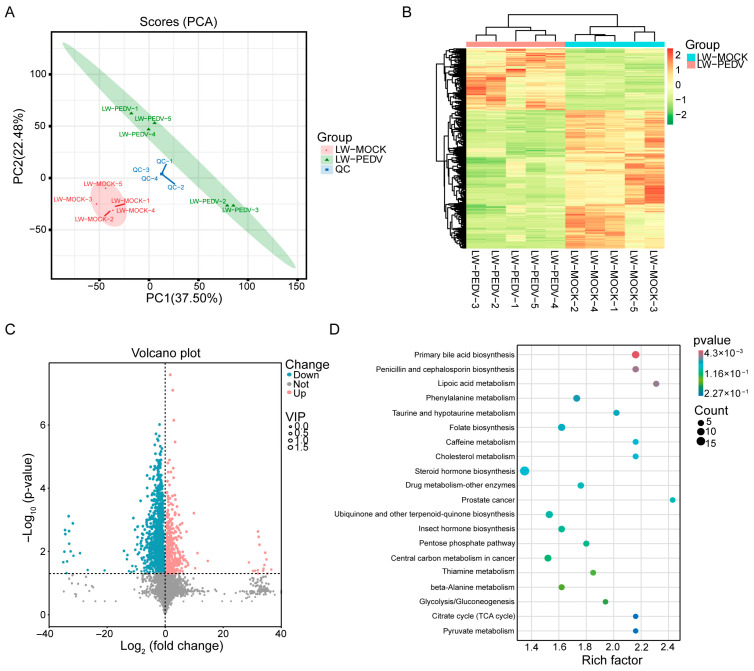
Metabolome analysis in mock and PEDV-infected groups. (**A**) Partial least square analysis of sample distribution based on identified metabolites. Red: mock group, green: PEDV-infected group, blue: quality control samples. (**B**) Hierarchical clustering analysis of differential metabolites between the mock group and the PEDV group. The color scale represents the Z-score normalized abundance of metabolites, ranging from green (low) to red (high). (**C**) Volcano plot of differential metabolites between two groups. Red and blue points represent significantly up-regulated and down-regulated metabolites, respectively. (**D**) KEGG pathway enrichment analysis of differential metabolites.

To explore the metabolic changes in PEDV-infected LW pigs, LC-MS/MS was used to carry out the metabolomics. PCA results indicated that the quality control (QC) was employed to analyze the repeatability of the samples and there was a significant difference between the mock group and the PEDV group (Figure 3A). Hierarchical clustering analysis described the expression patterns of differential metabolites between mock and PEDV groups (Figure 3B). Differential analysis identified 1485 differential metabolites, of which 459 were up-regulated metabolites and 1026 were down-regulated metabolites (Figure 3C, Table S4). KEGG analysis showed the pathways including ‘Primary bile acid biosynthesis’, ‘Lipoic acid metabolism’ and ‘Phenylalanine metabolism’ (Figure 3D, Table S5).

### 3.4. Integrated Analysis of Metabolome and Transcriptome Data in Pigs

**Figure 4:**
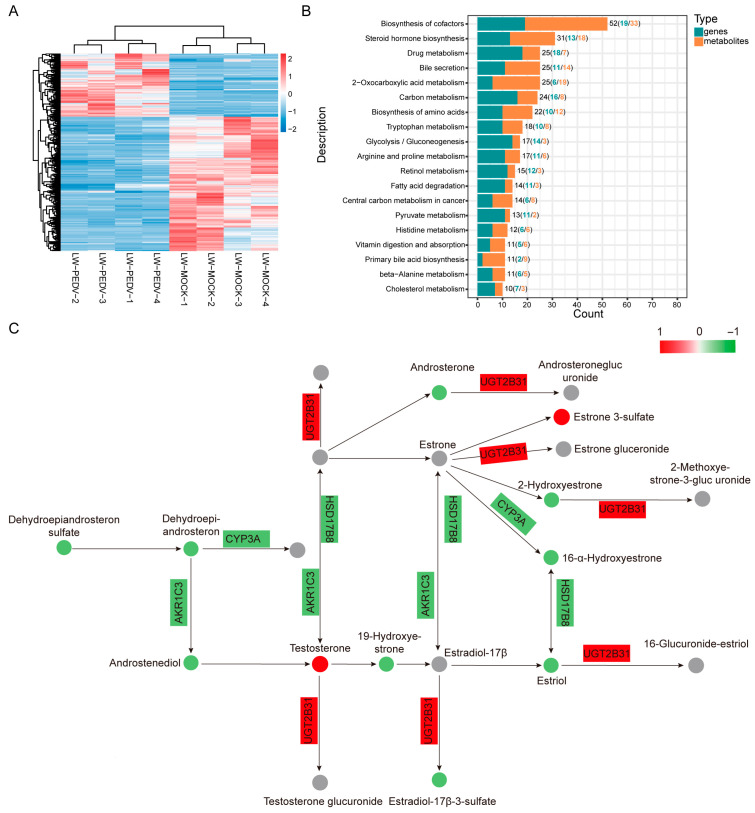
Integrated analysis of differential metabolites and DEGs between mock group and PEDV-infected LW pigs. (**A**) Hierarchical clustering heatmap of differential metabolites and differentially expressed genes. Blue represents downregulation and red represents upregulation. (**B**) KEGG pathway analysis of differential metabolites and differentially expressed genes. (**C**) Metabolites and genes involved in steroid hormone biosynthesis. Red dots: up-regulated metabolites, green dots: down-regulated metabolites, gray dots: undetected metabolites. Red frames: up-regulated genes, green frames: down-regulated genes.

To explore the correlation between differential metabolites and differentially expressed genes in PEDV-infected pigs, an integrated KEGG pathway enrichment analysis was carried out. Hierarchical clustering analysis revealed a strong correlation between differential metabolites and DEGs between two groups (Figure 4A). We conducted the KEGG enrichment analysis and found that differential metabolites and DEGs were co-enriched in pathways including ‘Steroid hormone biosynthesis’, ‘biosynthesis of cofactors’ and ‘Bile secretion’ (Figure 4B; Table S6). Notably, steroid hormone biosynthesis was one of the significantly enriched signaling pathways, indicating its potential role during PEDV infection in LW pigs (Figure 4C).

### 3.5. DHEA and Estriol Inhibited PEDV Infection in IPEC-J2 Cells

**Figure 5:**
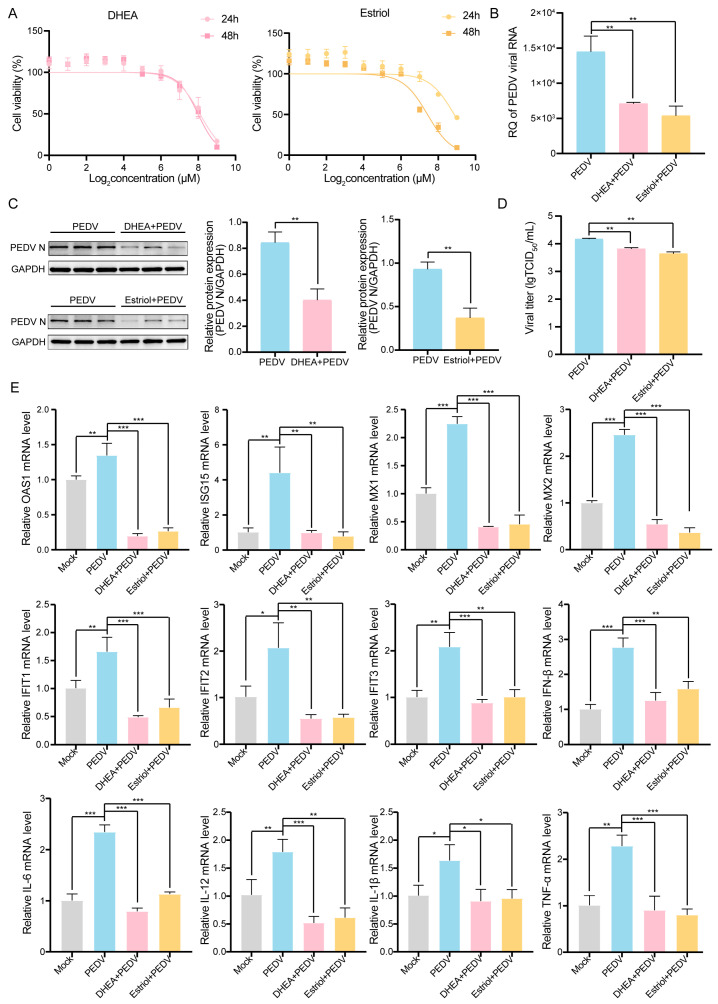
Effects of DHEA and Estriol addition on PEDV-infected IPEC-J2 cells. (**A**) Cell viability treated for 24 h or 48 h with DHEA or Estriol at different concentrations (1 µM, 2 µM, 4 µM, 8 µM, 16 µM, 32 µM, 64 µM, 128 µM, 256 µM, 512 µM). (**B**) PEDV M gene copy numbers in IPEC-J2 cells treated with 64 µM DHEA or Estriol at 24 h post-infection (hpi). (**C**) Expression of PEDV N protein. (**D**) The titers of PEDV in cells treated with DHEA or Estriol at 24 hpi. (**E**) Relative expression of antiviral ISGs and pro-inflammatory factors. Data are presented as the mean ± SD (n = 5). * *p* < 0.05, ** *p* < 0.01, *** *p* < 0.001.

Dehydroepiandrosterone (DHEA) has been reported to facilitate spontaneous viral clearance and immune control [22], while Estriol exerts therapeutic effects primarily through anti-inflammatory and immunomodulatory mechanisms [23]. To investigate the functional role of the steroid hormone biosynthesis pathway in PEDV infection, we assessed the effects of exogenous DHEA and Estriol supplementation on viral replication. We first assessed the cytotoxicity of DHEA and Estriol in IPEC-J2 cells using CCK-8 assay. The results indicated that 64 µM DHEA or Estriol was suitable for subsequent experiments (Figure 5A). qRT-PCR analysis indicated that DHEA and Estriol both decreased PEDV M gene copy numbers (Figure 5B). Furthermore, Western blot analysis showed that the expression of PEDV N protein was down-regulated by DHEA or Estriol treatment in PEDV-infected IPEC-J2 cells (Figure 5C). The titers of PEDV were also decreased with DHEA or Estriol added under PEDV infection (Figure 5D). In addition, the expression of antiviral ISGs (including OAS1, ISG15, MX1, MX2, IFIT1, IFIT2, IFIT3 and IFN-β) was significantly down-regulated following DHEA and Estriol treatment during PEDV infection (Figure 5E). Similarly, the expression of pro-inflammatory factors (IL-6, IL-12, IL-1β, TNF-α) was also decreased (Figure 5E). These results indicate that DHEA and Estriol not only inhibit viral replication but also alleviate the excessive inflammatory response and immune activation during PEDV infection.

## 4. Discussion

PEDV causes high mortality in suckling piglets, and the continuous emergence of variant strains has induced a significant economic loss to the global swine industry [24]. Vaccination is the main measure for PEDV prevention, but it offers limited protection [25,26]. Therefore, elucidating the mechanisms of host resistance is crucial for developing more effective intervention strategies. In this study, we utilized an LW pig challenge model to investigate the host response to PEDV. We first confirmed that PEDV infection induces severe histopathological changes, characterized by villous atrophy, shedding of mucosal epithelial cells, and degeneration of intestinal glands. To systematically understand the molecular mechanism underlying PEDV infection in vivo, we performed an integrated transcriptomic and metabolomic analysis of the small intestinal tissues. Our data constructed a comprehensive landscape of host–virus interactions, revealing distinct metabolic reprogramming events that may facilitate viral infection.

Transcriptome analysis is an effective tool to characterize the host’s cellular response to viral infections. Our study revealed the enrichment of GO terms including ‘positive regulation of DNA-templated transcription’ and ‘virion assembly’ with PEDV infection. It was reported that positive regulation of the DNA-templated transcription process was involved in the synthesis of the flock house virus and the metastasis of cancer, such as liver cancer [27,28]. Wu et al. found that the Ebola virus NP binds to the VP40 protein and then inhibits RNA synthesis and initiates virion assembly [29]. It was also reported that RNF5 interacts with the foot-and-mouth disease virus VP1 protein and inhibits virion assembly [30]. Therefore, we inferred that PEDV accelerates the synthesis of viral components required for progeny virion assembly via hijacking the transcriptional machinery in LW pigs. The enrichment of ‘lipoprotein metabolic process’, ‘NAD binding’ and ‘PPAR signaling pathway’ highlighted significant changes in lipid and energy metabolism. Peroxisome proliferator-activated receptors (PPARα, PPARβ/δ, PPARγ) belong to the nuclear receptor family of transcription factors and are activated by fatty acids and their derivatives [31]. The activation of PPAR promoted virus replication, such as the hepatitis B virus and the Zika virus [32,33,34]. It had been reported that PPAR signaling modulated the host immune response via inhibiting the release of cytokines [35]. As ligand-activated transcription factors, PPARs are key regulators of lipid and carbohydrate metabolism [36]. Hu et al. evaluated the effects of Atorvastatin ester in mice, indicating the regulatory role of PPAR signaling in lipid metabolism [37]. In this study, the activation of the PPAR signaling pathway suggested that PEDV may facilitate the synthesis of lipids and the activation of cytokine to promote PEDV replication in LW pigs.

Metabolomics offers an effective method to decipher the complex metabolic and potential mechanisms underlying virus infection. In our study, we found that PEDV infection mainly caused downregulation of metabolites, suggesting a rapid depletion of host nutrient reserves due to viral consumption. KEGG enrichment analysis revealed that three key metabolic pathways, including ‘Primary bile acid biosynthesis’, ‘Lipoic acid metabolism’, and ‘Phenylalanine metabolism’ were enriched in PEDV-infected pigs. Primary bile acid biosynthesis serves a dual function in supporting viral infectivity. Primary bile acids (PBAs) function as pivotal signaling molecules that inhibit pathogen colonization within the host or promote the replication of enteric viruses [38]. It had been reported that the taurocholic acid (TCA) restricts Chikungunya virus infection via modulating the bile acid–IFN signaling axis [39]. A study revealed that PBAs function as inhibitors of SARS-CoV-2 Nsp15 protein, thereby protecting against viral infection [40]. We therefore inferred that PEDV may dysregulate bile acid signaling pathways to facilitate viral entry in LW pigs. Lipoic acid (LA) plays a pivotal role in cellular bioenergetics by serving as a cofactor for dehydrogenation and oxidative decarboxylation processes [41]. Previous research has demonstrated that LA is essential for the defense against viral pathogens. For example, LA alleviated CVB3-induced myocarditis by fostering an immune response via neutrophil metabolic reprogramming [42]. We inferred that the enrichment of Lipoic acid metabolism in our study indicated a potential host defense strategy to modulate inflammatory response. Phenylalanine metabolism has been identified as a key regulator of host inflammation. For instance, high levels of phenylalanine aggravate acetaminophen-induced liver damage via promoting oxidative stress and modulating the MAPK pathway [43]. Chen et al. found that phenylalanine could inhibit mucosal repair enzymes and exacerbate intestinal barrier dysfunction [44]. In our study, the alteration of phenylalanine metabolism could contribute to PEDV pathogenesis.

Understanding the metabolic landscape of host–virus interactions is crucial for identifying novel therapeutic targets. It had been reported that chicoric acid inhibits PEDV attachment through remodeling of amino acid degradation pathways and PI3K-Akt signaling, as revealed by integrated transcriptomic and metabolomic analysis [45]. In our study, the most significant finding from our integrated multi-omics analysis is the convergence of transcriptional and metabolic alterations on the ‘Steroid hormone biosynthesis’ and ‘Bile secretion’ pathways. Bile acids function not only as facilitators of viral entry for enteric coronaviruses but also as critical modulators of the immune response. For instance, studies on SARS-CoV-2 and influenza have demonstrated that gut microbial composition and specific metabolites critically influence T-cell differentiation and cytokine responses [46]. A study on H1N1 influenza revealed that the virus could disseminate via the bloodstream to the liver and cause the downregulation of genes related to bile secretion and a marked decrease in secondary bile acids [47]. Similarly, our results indicate that PEDV infection triggers a profound perturbation in bile secretion. We inferred that this metabolic dysregulation not only facilitates viral pathogenesis but may also impair the host’s capacity to mount an immune response. Steroid hormone biosynthesis is involved in immune regulation, metabolic homeostasis, and epithelial barrier integrity [48]. It has been reported that Zika virus infection has been identified to induce a blockage in the steroid hormone biosynthesis pathway in vitro [49]. Furthermore, SARS-CoV-2 has been shown to directly alter steroidogenesis in critically ill patients, supporting that coronaviruses can directly impact host steroid metabolism [50]. In our study, the enrichment of steroid hormone biosynthesis after PEDV infection indicated that perturbation of steroid metabolism may represent the feature of PEDV infection. DHEA is a key downstream product of the steroid hormone biosynthesis pathway and alterations in this pathway lead to changes in DHEA levels [51]. Evidence from HIV research indicates that depleted DHEA levels are associated with adverse clinical outcomes, such as affective disorders in pregnant women [52]. It also had been reported that the antiviral efficacy of DHEA derivatives against flaviviruses was attributed to the specific inhibition of viral replication and RNA synthesis rather than viral entry [53]. Estriol serves as a critical downstream readout of the steroid hormone biosynthesis pathway, exhibiting high sensitivity to the host’s metabolic status [54]. A study on Influenza A virus demonstrated that exogenous Estriol treatment significantly improved disease outcomes by reducing pulmonary inflammation and suppressing the induction of proinflammatory cytokines and chemokines [55]. The protective role of Estriol against severe viral pathogenesis has been substantiated by systematic network analyses of COVID-19. It was demonstrated that estrogens interact with ESR1/2 receptors to significantly inhibit virus-induced inflammation and immune signaling [56]. In our study, exogenous DHEA and Estriol significantly inhibit PEDV infection. Furthermore, the concurrent downregulation of antiviral ISGs and pro-inflammatory factors following treatment suggests that these metabolites effectively restrict viral replication, thereby removing the stimulus for aberrant immune activation and promoting the resolution of inflammation.

This study identified significant dysregulation of steroid hormone biosynthesis during PEDV infection and established a strong correlation between these metabolic changes and viral replication using transcriptomic and metabolomic analyses. Notably, key metabolites DHEA and Estriol were identified to inhibit PEDV infection, while they also down-regulated antiviral ISGs and repressed the production of pro-inflammatory factors. However, the molecular mechanisms by which these metabolites exert their antiviral effects remain to be elucidated. Whether they act directly on viral particles, modulate the host’s cell membrane properties, or activate specific antiviral signaling pathways requires further investigation.

## 5. Conclusions

In conclusion, our study provides a comprehensive multi-omics landscape of the host response to PEDV infection in LW pigs. We demonstrate that PEDV infection not only causes severe intestinal pathology but also triggers systemic metabolic reprogramming. Most notably, integrated analysis highlights the convergence of transcriptional and metabolic dysregulation on the steroid hormone biosynthesis and bile secretion. Key metabolites in steroid hormone biosynthesis, DHEA and Estriol inhibit PEDV infection and induce the downregulation of antiviral ISGs and pro-inflammatory factors. These results indicate that PEDV strategically manipulates host hormonal homeostasis and bile acid metabolism to facilitate its pathogenesis. Our study expands the understanding of host–virus interactions and identifies specific metabolic pathways that could serve as novel targets for the development of antiviral therapeutics.

## Data Availability

The original contributions presented in this study are included in the article. Further inquiries can be directed at the corresponding author.

## Supplementary Materials

The following supporting information can be downloaded at: https://www.mdpi.com/article/10.3390/vetsci13040313/s1, Supplementary Tables S1–S7 (This file contains Supplementary Table S1 through Table S7). Supplementary Table S1 Differentially expressed genes between PEDV infection and mock groups in LW pigs. Supplementary Table S2 Gene ontology analysis for differentially expressed genes between PEDV infection and mock groups in LW pigs. Supplementary Table S3 Pathway analysis of differentially expressed genes between PEDV infection and mock groups in LW pigs. Supplementary Table S4 Differential metabolites between PEDV infection and mock groups in LW pigs. Supplementary Table S5 Pathway analysis of differential metabolites between PEDV infection and mock groups in LW pigs. Supplementary Table S6 Integrated pathways of differential metabolites and differentially expressed genes in LW pigs. Supplementary Table S7 The primer sequences of genes for qRT-PCR. Supplementary figure: Full original images of western blot.
