# Supplementary figures and images for: Integrated Metabolomic and Transcriptomic Analysis Reveals Host Response Mechanisms to Porcine Epidemic Diarrhea Virus Infection in Pigs
Source: Vet Sci. 2026 Mar 25;13(4):313. doi: 10.3390/vetsci13040313 (PMC13120392; doi:10.3390/vetsci13040313)

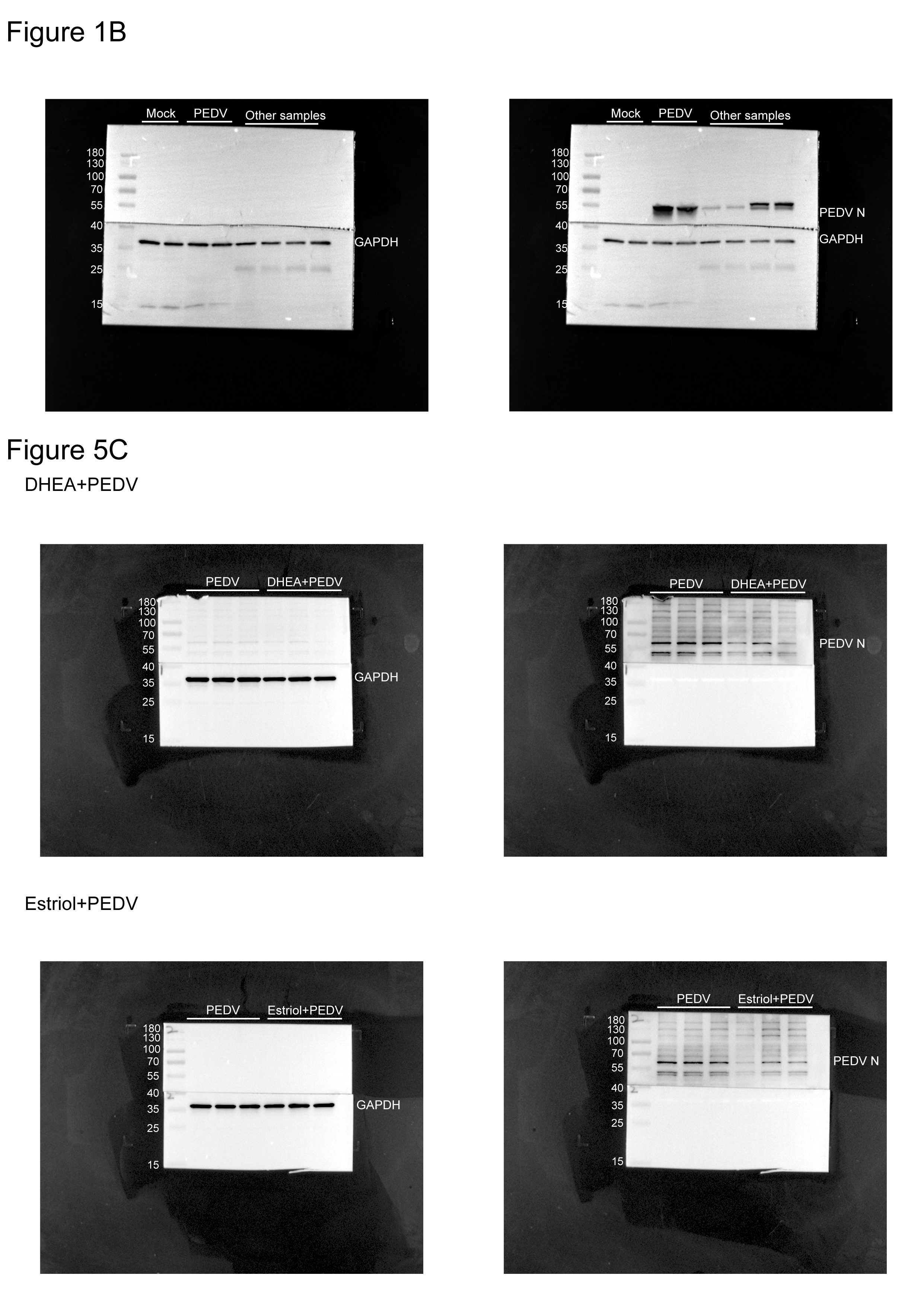

Supplement: Supplementary file 1 [file vetsci-13-00313-s001.zip › Full original images of Western blot.tif]
